# Hepatitis C Virus Hypervariable Region 1 Variants Presented on Hepatitis B Virus Capsid-Like Particles Induce Cross-Neutralizing Antibodies

**DOI:** 10.1371/journal.pone.0102235

**Published:** 2014-07-11

**Authors:** Milena Lange, Melanie Fiedler, Dorothea Bankwitz, William Osburn, Sergei Viazov, Olena Brovko, Abdel-Rahman Zekri, Yury Khudyakov, Michael Nassal, Paul Pumpens, Thomas Pietschmann, Jörg Timm, Michael Roggendorf, Andreas Walker

**Affiliations:** 1 Institute of Virology, University Hospital Essen, University of Duisburg-Essen, Essen, Germany; 2 Division of Experimental Virology, TWINCORE, Hannover, Germany; 3 Department of Medicine, Johns Hopkins University, Baltimore, Maryland, United States of America; 4 Virology and Immunology Unit, National Cancer Institute, Cairo, Egypt; 5 Centers for Disease Control and Prevention, Atlanta, Georgia, United States of America; 6 Department of Internal Medicine II, University Hospital Freiburg, Freiburg, Germany; 7 Department of Recombinant biotechnology, Latvian Biomedical Research and Study Centre, Riga, Latvia; Saint Louis University, United States of America

## Abstract

Hepatitis C virus (HCV) infection is still a serious global health burden. Despite improved therapeutic options, a preventative vaccine would be desirable especially in undeveloped countries. Traditionally, highly conserved epitopes are targets for antibody-based prophylactic vaccines. In HCV-infected patients, however, neutralizing antibodies are primarily directed against hypervariable region I (HVRI) in the envelope protein E2. HVRI is the most variable region of HCV, and this heterogeneity contributes to viral persistence and has thus far prevented the development of an effective HVRI-based vaccine. The primary goal of an antibody-based HCV vaccine should therefore be the induction of cross-reactive HVRI antibodies. In this study we approached this problem by presenting selected cross-reactive HVRI variants in a highly symmetric repeated array on capsid-like particles (CLPs). SplitCore CLPs, a novel particulate antigen presentation system derived from the HBV core protein, were used to deliberately manipulate the orientation of HVRI and therefore enable the presentation of conserved parts of HVRI. These HVRI-CLPs induced high titers of cross-reactive antibodies, including neutralizing antibodies. The combination of only four HVRI CLPs was sufficient to induce antibodies cross-reactive with 81 of 326 (24.8%) naturally occurring HVRI peptides. Most importantly, HVRI CLPs with AS03 as an adjuvant induced antibodies with a 10-fold increase in neutralizing capability. These antibodies were able to neutralize infectious HCVcc isolates and 4 of 19 (21%) patient-derived HCVpp isolates. Taken together, these results demonstrate that the induction of at least partially cross-neutralizing antibodies is possible. This approach might be useful for the development of a prophylactic HCV vaccine and should also be adaptable to other highly variable viruses.

## Introduction

At present, more than 180 million people worldwide are chronically infected with the hepatitis C virus (HCV). Despite many efforts (for review see [1]), there is still no vaccine against HCV. Only 30% of infected patients can spontaneously resolve the infection, and CD8^+^ T cells are the key component for this resolution [2]. However, neutralizing antibodies are also important in protecting people against HCV infection. Studies with HCV pseudoparticles (HCVpp) and cell culture-derived HCV (HCVcc) showed that neutralizing antibodies develop in spontaneous resolvers [3] and that rapid induction of neutralizing antibodies is associated with viral control [4], [5]. There is also evidence that intravenous drug users (IDUs) who have previously recovered from HCV infection are more likely than HCV-naïve IDUs to resolve the infection. Again, this resolution is associated with high titers of broadly neutralizing antibodies [6]–[8].

Given the importance of both cellular and humoral immune responses for protection against chronic HCV infection, a successful vaccine should be able to induce not only a vigorous T-cell response but also high titers of neutralizing antibodies capable of neutralizing various viral isolates. In HCV-infected patients, most neutralizing antibodies are directed against hypervariable regions I through III (HVRI–HVRIII) in envelope protein 2 (E2); therefore, these regions are a prime target antigen. Unfortunately, HVRI is also the most variable region of HCV, and its constant evolution allows the virus to escape the existing antibody response [9]. That sequence evolution is indeed driven by immune pressure is shown by the stability of HVRI in infected individuals with agammaglobulinemia [10], [11]. However, even in HVRI the sequence flexibility is not unlimited, because this region also contains highly conserved residues surrounded by mutational hotspots [12]. Furthermore, HVRI can be roughly divided into a highly variable N-terminal domain, which may serve as an immunological decoy [13], and a less variable C-terminal domain; the higher conservation probably reflects the functional importance of HVRI for the interaction with the SR-BI receptor and glycosaminoglycans and for shielding the CD81 binding site [3], [14]–[19]. Antibodies against this region are neutralizing [20], inhibit cell-to-cell spread in vitro [21], and protect chimpanzees from HCV challenge in vivo [22]. Recently, a phase I vaccination trial in humans with E1 and E2 as antigens showed that patient sera that were virus neutralizing contained high concentrations of high-avidity HVRI-specific antibodies [23]. However, in both studies protection was restricted to viruses containing the same HVRI sequences as those used for immunization. Hence, sequence heterogeneity in the HVRI remains an important challenge for HVRI-based vaccines.

One way of overcoming the problem of target heterogeneity is immunization with a combination of several HVRIs. First, these variants must be chosen in a way that enables them to induce a broad, cross-reactive and neutralizing antibody response. Second, the genuinely low immunogenicity of the HVRI peptides must be boosted, e.g., by combination with an immune-enhancing carrier. The first issue was recently addressed by the use of HVRI mimotopes [24]–[26] designed to be cross-reactive with many natural HVRI sequences and by selecting naturally occuring cross-reactive HVRI sequences from patient isolates [27]. Both types induce cross-reactive but genotype-specific antibodies. The second issue can be addressed by presentation of the peptides on capsid-like particles (CLPs). Viral capsids combine several features that make them attractive as antigen carriers for vaccine design [28]. The mammalian immune system is highly adapted to recognize particles of viral size (20–200 nm) [29] and viral appearance (e.g., repetitive surface, incorporated nucleic acids). The 183 aa hepatitis B virus (HBV) core protein (HBcAg) spontaneously assembles into icosahedral 34-nm particles [30]. Authentic nucleocapsids and *E. coli*–expressed CLPs are highly stable, can package nucleic acids, and are exceptionally immunogenic [31], [32]. B-cell epitopes, such as the immunodominant c/e1-epitope, are symmetrically arranged in repetitive arrays on the HBV CLP surface. This arrangement allows direct B-cell activation via B-cell receptor crosslinking [33]. In addition, HBV CLPs can induce both T cell–dependent and T cell–independent immune responses [32]. Finally, the much higher stability of the packaged versus free nucleic acid promotes its release only in late endosomes, where recognition via Toll-like receptors (TLRs) efficiently activates the innate immune system [34], [35]. This exceptional immunogenicity of CLPs can be transferred to foreign protein sequences, especially if they are inserted into the surface-exposed but sequence-internal c/e1 epitope of the core protein [36], [37]. In phase I trials, CLPs displaying small epitopes were immunogenic and well tolerated [38], [39]. Because of the central location of the insertion site, only small peptides or proteins with a compatible structure or size can be presented on HBV CLPs without disturbing their structure and self-assembling capability [40]–[42]. We recently overcame this limitation by splitting the core protein inside the c/e1 loop into two self-complementing fragments (CoreN and CoreC) that associate into regularly shaped CLPs (SplitCore system). Foreign sequences can now be fused to CoreN, CoreC, or both without imposing conformational stress, largely regardless of their structure and size [43]. Importantly, the surface orientation of the antigen can be deliberately chosen depending on whether it is fused to CoreN or CoreC.

In the present study, we used this system with the goal of showing that the presentation of HVRI variants on SplitCore CLPs boosts the immune response and that a selected number of HVRI variants can induce broadly neutralizing antibodies.

## Results

### Design and expression of recombinant HVRI core fusion proteins

**Figure 1:**
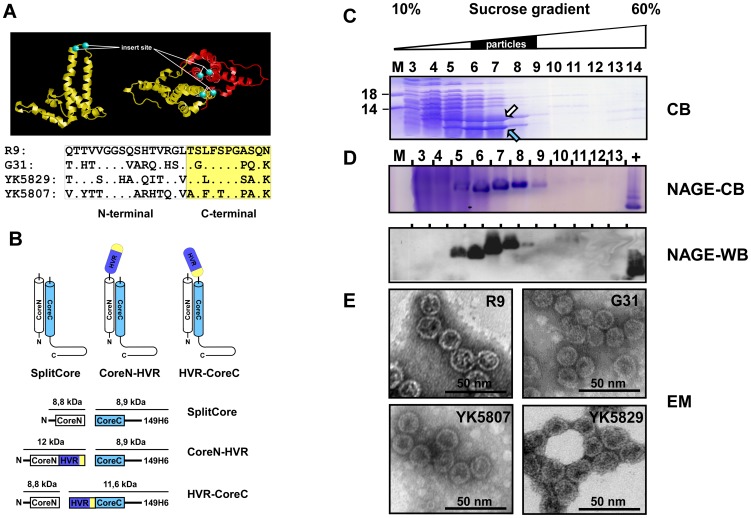
Efficient particle formation of HVRI SplitCore fusion proteins. **A** X-ray structures of HBV core protein. Side and top views of the HBc monomer (PDB, 1QGT; aa 1–143). The insertion site for HVRI variants lies in the c/e1 epitope connecting helix α-3 and helix α-4. In SplitCore constructs the core protein was separated between residues Pro-79 and Ala-80 (green) spheres, indicating C-positions. Below: aa sequence of the HVRI variants used. The C-terminal part is marked yellow. **B** Schematic view. The antigen orientation on the particle surface can be deliberately chosen depending on the fragment to which the HVRI variants are fused. Fusion of HVRI variants to the CoreN fragment exposes the more conserved C-terminus of the HVRI. Linker sequences (G_4_S) are not shown. **C** Expression and particle formation of CoreN-HVRI-R9. Crude lysate from the bacteria-expressing CoreN-HVRI-R9 fusion protein was sedimented through a preparative 10% to 60% sucrose step gradient; 14 fractions of 860 µl each were harvested from the top. Aliquots of 8 µl each were analyzed by SDS-PAGE and Coomassie Blue (CB) staining; marker proteins with their molecular masses (in kDa) are indicated on the left. Both fragments, CoreN (arrow up) and CoreC (arrow down), peaked around fractions 5 to 8, as is typical for intact CLPs [77]. **D** Native agarose gel electrophoresis (NAGE). Aliquots of the gradient shown in C were run in 1% agarose gels; they were either stained with CB or the gel content was blotted onto polyvinylidene difluoride (PVDF) membranes and particles were detected with the particle-specific mAb 3120 [78] **E** Electron microscopy. Aliquots of the fusion proteins were negatively stained with uranyl acetate.

Because of the huge intragenotype differences, we used HVRI variants that were most similar to genotype (GT) 1b for proof of principle that HVRI-displaying CLPs can induce neutralizing antibodies. The sequence of the four 27 aa long HVRI variants is depicted in Figure 1. Variants YK5807 and YK5829 are naturally occurring cross-reactive HVRI variants derived from patients infected with HCV GT1b (Fig. 1A). Variants R9 and G31 are artificially selected HVRI mimotopes generated by Puntoriero et al. [44]. The mimotope R9 is similar to Gt1b and was reported to react with 75% of patient sera. Although the mimotope G31 is similar to GT3a, it was included because of its high cross-reactivity (79%) and because we wished to determine whether an HVRI variant from a different genotype can broaden the response.

Fusion of the HVRI sequences to the SplitCore CoreC fragment (Fig. 1B) would mimic the natural orientation of the HVRI in the E2 protein (exposed HVRI N-terminus). However, because the neutralizing determinants are believed to be located primarily in the C terminus [45], [46], we decided to fuse the HVRI variants to the CoreN fragment to maximize its exposure to the immune system.

The various fusion proteins were highly expressed in *E. coli* (as high as 10–15 mg/L of culture) and soluble. For the SplitCore HVRI R9 fusion protein, the CoreN HVRI and the CoreC fragment co-sedimented into particle-typical center fractions in sucrose gradients (fractions 5–8 of 14) (Fig. 1C), indicating that SplitCore HVRI R9 can form CLPs. In the same gradient fractions, assembled particles were detected by native agarose gel electrophoresis (NAGE), where particles with their low diffusion coefficient migrate as distinct bands (Fig. 1D NAGE-CB). Immunostaining with the HBcAg particle-specific monoclonal antibody (mAb) 3120 further confirmed the formation of particles (Fig. 1D NAGE-WB). Comparable data were obtained for the other three fusion proteins (not shown). Finally, electron microscopic inspection of all four HVRI CLPs showed abundant spherical particles (Fig. 1E). As expected from the relatively small size of the fused HVRI sequences, no morphological differences were detectable between HVRI CLPs and wild-type (wt) CLPs.

To determine whether immunization with a single HVRI variant is sufficient to induce neutralizing antibodies, we first used each purified HVRI CLP preparation separately for immunization. C57BL/6 mice were immunized subcutaneously on days 0, 14, 28, and 56 with 20 µg of SplitCore fusion proteins HVRI R9, HVRI G31, HVRI YK5809, or HVRI YK5829. Antibody titers were determined on days 28, 56, and 84.

**Figure 2:**
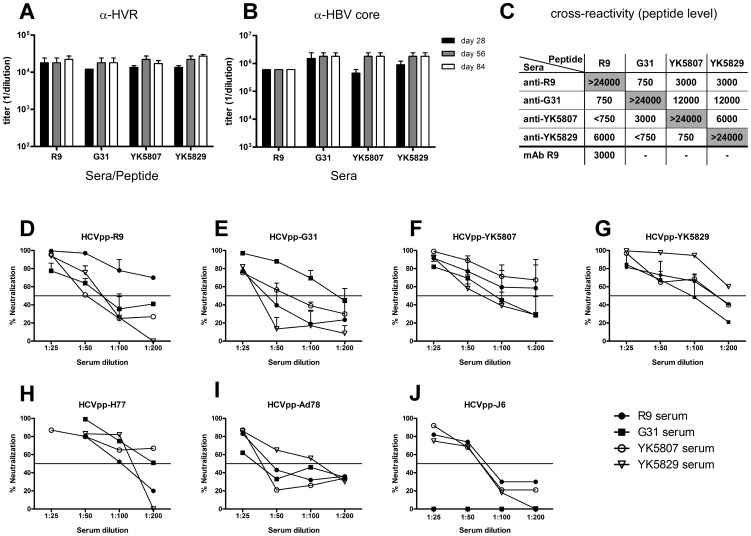
HVRI CLPs are highly immunogenic in mice and induce neutralizing antibodies. Groups of mice (6 per group) were immunized subcutaneously with the four HVRI particles (20 µg per variant) in IFA at days 0, 14, 28, and 56. Mice were bled on days 0, 28, 56, and 84. Serum was pooled and tested for HVRI peptide and HBV core-specific antibodies by ELISA. Bars represent the mean of 3 replicate values; error bars represent standard error of the mean (SEM). **A** Antibody response against the HVRI variant used for immunization. **B** Antibody response against HBcAg. **C** Cross-reactivity between the various HVRI variants. A monoclonal antibody against R9 (concentration, 1.2 µg/µl) was used as a control. **D–J** HCVpp encoding a luciferase reporter were preincubated with pre-sera or with day 84 sera for 2 h before infection of Huh-7.5 cells. Luciferase activity was measured 72 h after infection. Neutralization efficacy was calculated by comparing the infectivity in the presence of preimmune serum to the infectivity in the presence of postimmune serum. Values higher than 50% were scored as neutralization (continuous black line).

After two immunizations, all constructs induced high titers of HVRI-specific antibodies (endpoint titers in the range of 1∶15,000 to 1∶24,000; Fig. 2A). After four immunizations, all HVRI CLPs induced final titers higher than 1∶24,000. As expected, all sera also contained high titers of carrier-specific antibodies (anti-HBc; Fig. 2B), demonstrating the excellent immunogenicity of the core protein. The antigenic cross-reactivity between the HVRI variants was determined in antisera from day 84 (Fig. 2C); the corresponding peptides were used as targets. A monoclonal antibody directed against the R9 sequence, which was used as a control, reacted exclusively with the R9 peptide. As expected, all CLP-induced sera reacted strongly to the HVRI variant used for immunization (highest dilution tested, 1∶24,000; Fig. 2C, shaded in grey); however, some of the heterologous peptides were also well recognized, even though none of the sera reacted with all test peptides. Hence, the HVRI CLPs induced at least a partially cross-reactive antibody response.

### Anti-HVRI antibodies cross-neutralize chimeric HCVpp

To analyze the neutralization ability of our antisera, we generated chimeric HCV pseudoparticles (HCVpp) in which the HVRI-encoding region of the E2 protein from the HCV single-source outbreak AD78 isolate ([4], Gt1b) was swapped for the corresponding R9, G31, YK5807, or YK5829 sequences (HCVpp-R9, -G31, -YK5807, and -YK5829, respectively). Replacement of the HVRI had little effect on HCVpp formation, and all chimeric HCVpp formed infectious particles. As expected, all antisera were able to neutralize the HCVpp containing the HVRI variant used for immunization in a dose-dependent manner (neutralization at dilution, 1∶200: R9, 70%; G31, 41%; YK5807, 67%; and YK5829, 60%; Fig. 2D–G). Cross-neutralization of the other HCVpp was detectable but clearly weaker (Fig. 2D–G). Interestingly, although all antisera efficiently neutralized HCVpp containing the natural HVRI variants (YK5807 and YK5829), HCVpp with HVRI mimotopes (HCVpp-R9 and HCVpp-G31) were neutralized only by R9 antisera or G31 antisera, respectively. To rule out the possibility that the observed cross-reactivity is biased by our choice of the four HVRI variants, we further analyzed neutralization of HCVpp bearing the H77c ([17], Gt1a), AD78 ([4], Gt1b), or J6 ([47], Gt2a) sequence (HCVpp-H77, -Ad78, and -J6, respectively) (Fig. 2H–J). At a dilution of 1∶100 all antisera were able to neutralize HCVpp-H77 with 50% to 80% efficiency. Neutralization was still detectable at a dilution of 1∶200 in case of anti-YK5907 and anti-G31 antibodies (Fig. 2H). HCVpp-Ad78 was neutralized by anti-YK5829 antibodies up to a dilution of 1∶100 but not by R9-, G31-, or YK5807-specific antibodies (Fig. 2I). Interestingly, HCVpp-J6, containing a genotype Gt2a HVRI, was poorly neutralized by all antisera, and G31 anti-serum completely failed to react with HCVpp-J6 in several independent experiments (Fig. 2J). In conclusion, immunization with even one HVRI variant induced strong and partially cross-neutralizing antibodies.

### Combination of HVRI CLPs induces cross-reactive antibodies

**Figure 3:**
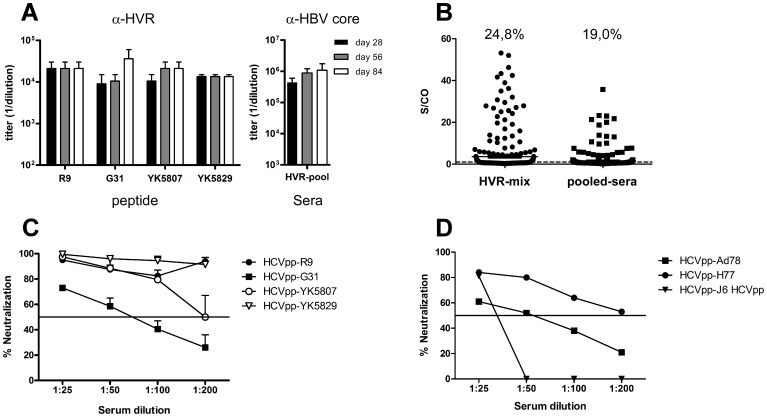
Immunization with pooled HVRI CLPs enhances cross-reactivity. **A** Mice were immunized subcutaneously with a mixture of HVRI CLPs (5 µg per variant) in IFA, and sera were analyzed as described in Fig. 2. **B** Reactivity with 326 patient-derived HVRI peptides. Sera from day 84 after immunization with mixture HVRI CLPs (HVRI mix) and pooled sera from a single immunization were tested with ELISA at a dilution of 1∶800 for binding to 326 naturally occurring patient-derived HVRI peptides. Peptides yielding S/Co >1 were scored as positive. The cut-off value was the optical density at λ = 495 nm+3.5*StDev obtained with unrelated peptides. **C** Neutralization of HCVpp bearing HVRI variants used for immunization. **D** Neutralization of unrelated heterologous HCVpp. For details see Fig. 2.

Aiming to further improve the neutralization capability, we vaccinated mice with a mixture of all four HVRI CLPs simultaneously (5 µg per construct, termed HVRI mix). Immunization with HVRI mix induced a strong HVRI-specific antibody response (Fig. 3 A, left, endpoint titer of 1∶14,000 to 1∶36,000), cross-reacting with peptides corresponding to all four HVRIs used for immunization. Anti-HBc-specific antibody titers were comparable to those detected after immunization with the single HVRI CLPs (Fig. 3 A, right).

To analyze the cross-reactivity with naturally occurring HVRI sequences, we used ELISA to test the binding of HVRI mix sera to a panel of 326 patient-derived HVRI peptides from various genotypes. For comparison we used pooled sera from immunization with single HVRI CLPs (termed pooled sera). The HVRI mix sera reacted with 81 of 326 peptides (24.8%) while the pooled sera reacted with 62 of the 326 peptides (19%) (Fig. 3B). Compared to pooled sera, the HVR mix sera also showed a stronger binding of individual peptides (Fig. 3B).

The increased cross-reactivity after immunization with HVRI mix was also reflected by enhanced neutralization of HCVpp. HVRI mix–specific antibodies neutralized HCVpp-R9, -YK5807, -YK5829, and HCVpp-H77 up to a dilution of 1∶200 (Fig. 3C). Interestingly, HVRI mix antisera were also poorly able to neutralize HCVpp-J6 (Fig. 3D, black triangles), a finding indicating that immunization with HVRI mix induced a strong and broad antibody response but was not cross-reactive with genotype 2a.

### AS03 adjuvant further boosts the immunogenicity of HVRI CLPs

**Figure 4:**
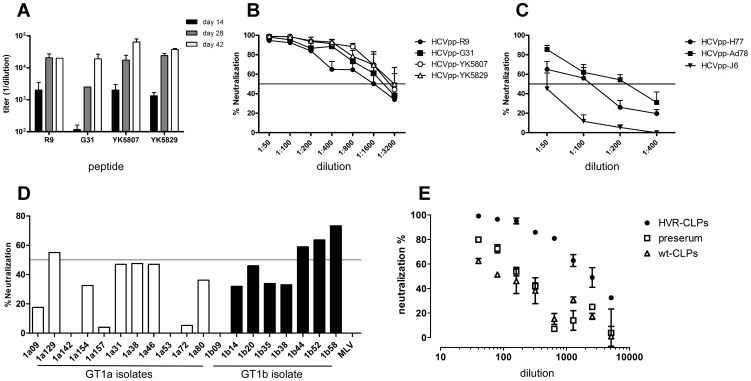
The water-in-oil adjuvant AS03 increases antibody and neutralization titers. **A** Mice were immunized intramuscularly with pooled HVRI CLPs (5 µg per variant) in AS03 on days 0, 14, and 28. Mice were bled on days 0, 14, 28, and 42. Sera were analyzed as described in Fig. 2 **B** Neutralization of homologous HCVpp. **C** Neutralization of heterologous HCVpp. For details see Fig. 2. **D** Neutralization of patient-derived HCVpp. Mice sera from day 42 were tested at a dilution of 1∶50 for the ability to neutralize patient-derived HCVpp from GT1a isolates (white bars) and GT1b isolates (black bars). Continuous line: 50% neutralization. HVRI sequences of the HCVpp are depicted in Table S1. **E** HVRI CLPs can induce HCVcc-neutralizing antibodies. Huh7.5 cells were inoculated with JC1-YK5829 viruses in the presence of decreasing amounts of serum from day 42. Pre-serum and serum from mice immunized with wild-type CLPs served as a control. Neutralization efficiency was determined by luciferase assays 72 h after inoculation. Data are expressed as means and standard deviations of triplicate measurements. One of three representative experiments is shown.

The previous immunizations were performed with water-in-oil adjuvant IFA as an adjuvant. To determine whether an oil-in-water adjuvant formulation could further improve immunogenicity, a mixture of all four HVRI CLPs were administered in AS03 in a 14-day immunization scheme (termed AS03-M). Although anti-HBc–specific antibody titers were comparable to those achieved in the previous immunizations, AS03-M induced a much stronger HVRI-specific antibody response than did HVR CLPs in IFA (Fig. 4 A; endpoint titer of 1∶32,000 to 1∶80,000). Furthermore, the neutralization capability of AS03-M antisera was dramatically increased, especially against HCVpp-R9, -G31, -YK5807, and HCVpp-YK5829, all of which were neutralized up to a dilution of 1∶3,200 (Fig. 4B). The neutralization of HCVpp-H77 and HCVpp-AD78 was also enhanced (Fig. 4C). However, in line with previous findings, J6-HCVpp was not neutralized (Fig. 4C, black triangles).

### HVRI CLP–induced antibodies can neutralize patient isolates

For proof of principle that antibodies induced by AS03-M are broadly neutralizing within one HCV genotype, we tested the neutralization of 19 additional HCVpps from genotypes 1a and 1b [5]. When the cutoff for neutralization was set to 50% the AS03-M serum was able to neutralized 4 of 19 (21%) HCVpp (**Figure 4**D). Interestingly, when the cutoff is slightly reduced to 45% the AS03-M serum was able to neutralize 8 of 19 (42%) HCVpp (Fig. 4D), indicating that with slight lower dilutions more HCVpp could be neutralized. As expected, HCVpp from GT1b were neutralized more efficiently; however, selected HCVpp from GT1a were also neutralized. Interestingly, the only sequence similarities between the neutralized HCVpp are aa 20–24 in the C-terminal part of the HVRI (Table S1).

Finally, we analyzed the ability of the anti-HVRI antibodies to neutralize infectious JC1-HCVcc virions (a genotype 2a chimera, [47]). No antiserum was able to neutralize JC1. This finding was expected because neither the J6-HVRI peptide nor the HCVpp-J6 (both represent the HVRI present in JC1) was recognized by our HVRI-specific antibodies. Therefore, we replaced the J6-HVRI in the JC1 virus with the naturally occurring HVRI variant YK5829. Incubation of JC1-YK5829 with AS03-M sera efficiently blocked the infection of Huh7.5 cells in a dose-dependent matter (Fig. 4E), whereas pre-serum or serum from mice immunized with empty wt CLPs did not. A JC1 variant lacking the HVRI region was not neutralized (not shown), showing that neutralization was HVRI dependent.

Taken together, we could show that immunization with 4 designed HVRI variants presented on CLPs induces high titers of cross-reactive antibodies that can neutralize many circulating GT1a and GT1b isolates.

## Discussion

HCV infection is still a fundamental health problem, especially in developing countries. Although new antiviral therapies are rapidly developed and promise to effectively cure most of the infected patients in the developed world, a protective vaccine would be needed for the eradication of HCV. An effective vaccine should elicit broad immune responses conferring antibody- and cell-mediated protection [4], [6], [20]. In particular, the induction of strong and broadly neutralizing antibody responses against HCV is a challenging task. Vaccination trials in humans with recombinant E1 and E2 showed that a key determinant in neutralizing serum samples is the presence of high-affinity HVRI-specific antibodies [23]. Previous vaccination with various HVRI peptides induced HVRI-specific binding antibodies [24], [25], [44]; however, their neutralizing potential was not addressed. Our current findings showed that immunization with CLPs presenting HVRI variants can indeed induce neutralizing antibodies. We further showed that immunogenicity is improved by using SplitCore CLPs as particulate antigen carriers and by deliberate exposure of the conserved parts of HVRI on the particle surface.

The first important observation was that HVRI SplitCore fusion proteins formed intact CLPs. Genetic insertion of heterologous sequences into surface-exposed loops of viral capsid proteins often suffers from structural incompatibility and inaccessibility of neutralizing epitopes. The HBc SplitCore approach overcomes this restriction by providing surface-exposed N- and C-termini, which leads to the successful presentation of HVRI variants on intact particles. Structural integrity of the HVRI CLPs is crucial because enhanced immunogenicity is intimately linked to intact particles [29]. Although large aggregates can also induce a strong immune response [48], intact particles induce higher titers and, in particular, more neutralizing antibodies [43]. Our HVRI CLPs induced high titers of antibodies comparable to those induced by multiple antigenic HVRI peptides [25], and our antibodies were highly neutralizing. Taken together, the findings of the present study corroborated the observation that the presentation of small peptides on a large antigen carrier enormously enhances their immunogenicity [43], [48]–[51].

Another advantage of SplitCore CLPs is the expression in bacteria. Bacterial expressed CLPs contain only bacterial mRNA [31], avoiding any potential regulatory concern about eukaryotic or viral DNA content in the CLPs. However, during assembly the CLPs can package *E.coli* proteins and crude preparations usually contain high concentrations of endotoxin. The later issue has been solved by repeated TX114 phase separation reducing the endotoxin concentration >1 EU/mg [52]. To purify CLPs from packed protein, CLPs can be disassembled into dimers, purified and later reassembled into CLPs [53].

It may be noteworthy that HBcAg is derived from a human pathogen and that worldwide approximately 2 billion people are anti-HBcAg positive. The role of preexisting immunity to a carrier is still controversial, with examples in favor of [54], [55] and against [49] carrier-mediated suppression [56]. Although repeated vaccinations with HVRI-CLPs did not lead to carrier-mediated suppression of the anti-HVRI response, despite the strong anti-carrier response, the use of HBcAg as a carrier may therefore be problematic. This potential shortcoming can be overcome by substituting HBcAg with other hepadnaviral core proteins [43], [55], [57], [58]. Woodchuck hepatitis core protein (WHc), for instance, is not cross-reactive with HBcAg-specific antibodies. Indeed, the mimotope R9 fused to a split woodchuck core variant (SplitWHc-R9) efficiently formed CLPs (Fig. S1) In previous studies, WHc CLPs and HBc CLPs were comparable in immunogenicity [55], [58].

Because of the enormous variability of HCV, the induction of highly cross-reactive antibodies is very important for an effective vaccine. Although we used primarily HVRI variants similar to GT1b for immunization, the HVRI mix antibodies were cross-reactive with 25% of patient-derived HVRI peptides from all genotypes. In the HCVpp neutralization assay we observed neutralization of Gt1b HCVpp and also of GT1a HCVpp. Notably, the only sequence similarity between the neutralized HCVpps are 4 aa located in the C-terminal part of HVRI. This finding agrees with those of a previous study [45] and highlights the importance of the correct antigen orientation on a carrier [43].

Rey et al showed that high-affinity HVRI-specific antibodies are a key determinant of neutralization [23]. Therefore, one of the most important observations was that immunization by HVRI CLPs with AS03 adjuvants induced antibodies with drastically enhanced neutralization efficiency. As shown previously, the TH1 response induced by AS03 may have led to the production of superior neutralizing antibodies [59]. The enhanced stability of the particles in the oil-in-water emulsion AS03 may be another explanation for the enhanced neutralization. Intact particles are crucial for efficient B-cell activation and maturation, and both the core protein and the assembled particle structure are stabilized by hydrophobic interactions [60] and may therefore be more stable in AS03.

The antibodies were also able to inhibit the infection of Huh7.5 cells with HCVcc-derived viruses but only when the GT2a HVRI was swapped with a GT1 HVRI. This finding was expected because we used GT1b HVRI sequences for immunization, and the antibodies were not able to neutralize GT2a-derived HCVpp and HCVcc (HCVpp-J6 and HCVcc, respectively). Interestingly, Meunier et al. [61] also observed good cross-reactivity within GT1 and a lack of cross-reactivity with GT2 and GT3 when they immunized chimpanzees with complete E1/E2 from GT1a. This finding implies that HVRI variants from all genotypes must be included in a vaccine cocktail. And, indeed, preliminary findings suggest that immunization with a number of selected HVRI peptides induces cross-reactive antibodies that react with more than 95% of our peptide library [27]. Using twenty or more related antigens in a vaccine cocktail is a feasible approach, as shown by the commercial 23-valent polysaccharide vaccine, which has been used for 35 years in the vaccine against *Streptococcus pneumoniae* [62].

The exact nature of protective HCV immunity is not clear. Compelling evidence suggests that self-limiting infection is associated with an early anti-HCV antibody response [4], [6], [20]. However, the role of neutralizing antibodies in the late phase of infection remains elusive, because most chronically infected patients cannot clear the infection despite the presence of neutralizing antibodies [3], [63]–[65]. Interestingly, immunizing chimpanzees with complete E1/E2 protected 4 out of 5 animals. The one animal with viral breakthrough developed an acute self-limiting infection [61]. These findings lead us to speculate that neutralizing antibodies in the early phase attenuate the HCV infection. Because of the enormous number of HCV quasispecies, it is unlikely that an HVRI-based vaccine will offer sterile protection. Rather, such antibodies may blunt the HCV infection to facilitate the development of protective cellular immune responses [66]. Therefore, the most promising vaccine should be a combination of HVRI CLPs and vaccines that induce robust T-cell responses, such as transgenic adenoviruses or MVA [67]. For example, Barnes et al. [68] have recently shown that immunization with adenoviral vectors encoding the HCV NS genes induces strong, broad, and long-lasting T-cell responses in humans. The authors suggest that such a vaccine should accelerate the generation of HCV immunity after infection. At the same time, Barouch et al. showed in the SIV model that immunization with an adenovirus primer and a MVA boost is partially protective; however, a substantial reduction in the risk of infection requires the inclusion of envelope proteins in the vaccine [69].

In conclusion, by presentation of HVRI-peptides in a top-side down orientation on SplitCore CLPs we were able to induce cross-protective antibody in mice. This approach might be useful for the development of a HCV vaccine, however could also be adapted for other highly variable pathogens.

## Materials and Methods

### Ethical statement

Mice entered experiments when they were between 8 and 10 weeks of age and treated in accordance with the 8th Edition Guide for the Care and Use of Laboratory Animals and the institutional guidelines of the University Hospital Essen, Germany. The study was approved by the Northrhine-Westphalia State Office for Nature, Environment and Consumer Protection (LANUV NRW) and was carried out on the project license numbers G1088/09 and G815/05 issued by the same state office. The animals were housed under specific pathogen free [SPF] conditions in ICV mouse racks. All experiments were performed under isoflurane anesthesia. Mice were sacrificed by cervical dislocation under isoflurane anesthesia and all efforts were made to minimize suffering.

### Plasmid constructs

All fusion protein constructs were based on the parental plasmids pET28a2_HBc_c1-79_80-149H6 [43] and featured a T7 promoter–controlled synthetic [70] HBV SplitCore protein (HBV genotype D, serotype ayw; accession no.: CAA24706) truncated after aa149 and containing a C-terminal His6 tag. HVRI variants were introduced by standard PCR mutagenesis using long primers encoding the complete HVRI variants including a G4S linker. Plasmid pNL4-3.luc.R-E and phCMV-IRES-E1-E2 (H77) were kindly provided by Jane McKeating. Plasmid phCMV-IRES-E1-E2 (Ad78) was kindly provided by Thomas Baumert. HCVpp derivatives were generated by introducing the corresponding HVRI sequence by standard PCR mutagenesis into plasmid phCMV-IRES-E1-E2 (Ad78). All constructs were verified by DNA sequencing.

### Protein expression and purification

*E. coli* BL21(DE3) Codonplus cells (Stratagene) were used throughout as described [43], [71], [72]. Expression and purification were performed as previously described [42]. In brief, crude *E. coli* lysates were loaded onto 10% to 60% sucrose step gradients and sedimented at 20°C. Depending on the sample volume, we used SW28 rotor (3∶45 h at 28,000 rpm) or SW40 rotor (2 h at 40,000 rpm). The gradients were harvested in 14 equal fractions from the top (2.6 ml per fraction for SW28; 820 µl per fraction for SW40). For further purification, the pooled center gradient fractions were dialyzed against TN150 buffer (25 mM Tris-Cl [pH 7.5], 150 mM NaCl), concentrated, and subjected to a second gradient sedimentation or size exclusion chromatography on Superose 6 (GE Healthcare).

### Endotoxin

Endotoxin was depleted by Triton X-114 phase separation and repeated dialysis against excess PBS at 4°C, as described previously [43]. Endotoxin contents dropped from approximately 10^4^ EU/µg protein in the crude lysates to less than 1 EU/µg, as determined by the pyrochrome assay (Associates of Cape Cod, Inc., MA).

### Native agarose gel electrophoresis (NAGE) and immunoblotting

NAGE was performed in 1% agarose gels, as previously described [31]. For immunological detection, NAGE or SDS-PAGE gels were blotted to PVDF membranes, which were probed with specific primary antibodies and peroxidase-conjugated secondary antibodies plus chemiluminescent substrates. We used the monoclonal anti-HBc antibodies 10E11 and 10F10 (anti-coreN and anti-coreC, respectively) [73] and the particle-specific 3120 [30].

### Electron Microscopy

Negative staining electron microscopy (EM) using 2% uranyl acetate was kindly performed by G. Lattwig (Pathology, University Hospital Essen), as previously described [74].

### Immunization experiments in mice

Groups of 6 to 10 female C57BL/6 mice (Harlan Laboratories, Germany) housed under specific pathogen–free (SPF) conditions were immunized with HVRI CLPs either intramuscularly (i.m.) with AS03 or subcutaneously (s.c.) with incomplete Freund's adjuvant (IFA). Mice were immunized with either 20 µg of single HVRI CLPs or 20 µg of HVRI mixture (5 µg per variant) as indicated. Mice were bled either at days 28, 56, and 84 or at days 14, 28, and 42. Sera were pooled, and anti-HBc or anti-HVRI antibodies were measured by indirect solid-phase ELISA using solid-phase wt HBcAg (50 ng per well) or HVRI peptides (0.5 mg per well). Goat anti-mouse IgG (or IgG isotype-specific) antibodies were used as secondary antibodies. Synthetic peptides were synthesized by the CDC peptide synthesis facility (Atlanta, Georgia, US). Peptide sequences used for cross-reactivity analysis can be obtained upon request.

### HCV pseudoparticles (HCVpp) and HCVcc neutralization experiments

Human immunodeficiency virus (HIV)-HCV pseudotypes were generated as previously described [17]. Briefly, we used the CalPhos mammalian transfection kit (Clontech) to co-transfect 2.5×10^6^ 293T cells with plasmid pNL4-3.luc.R-E [75] encoding HIV ΔEnv and expressing luciferase or with vector phCMV-IRES-E1-E2 [76] encoding the HCV envelope glycoproteins. Sixteen hours after transfection, the medium was replaced. Supernatants were harvested at 48 h and 72 h, pooled, and clarified by centrifugation. The resulting supernatants were immediately used for infectivity and neutralization assays.

To investigate pseudotype virus infectivity, we seeded 5×10^5^ Huh7.5 cells in 96-well plates on the day before infection. The infection medium was removed, and HCVpp diluted in DMEM supplemented with 6% FCS were added. For neutralization, serum samples were mixed with the HCVpp at various dilutions and were incubated at 37°C for 2 h before to infection of the cells. After 72 h of incubation, the medium was removed, and cells were lysed with Bright Glo lysis buffer (Promega) for 2 h at −20°C. Luciferase activity was measured 10 min after the addition of the Bright Glo Luciferase Assay buffer (Promega) in a luminometer (Glomax Multi Detection System, Promega). Cell lysates were measured in triplicate; data are expressed as the mean of the results from at least two independent experiments. HCVcc neutralization experiments were performed as previously described [14].

## Supporting Information

Figure S1 **Expression and particle formation of SplitWHC-R9.** **A** Crude lysate from bacteria-expressing SplitWHC-R9 fusion protein was sedimented through a preparative 10% to 60% sucrose step gradient; 14 fractions of 860 µl each were harvested from the top. Aliquots of 8 µl each were analyzed by SDS-PAGE and Coomassie Blue (CB) staining; marker proteins with their molecular masses (in kDa) are indicated on the left. Both fragments, CoreC (arrow up) and CoreN-R9 (arrow down), peaked in the center fractions. **B** Native agarose gel electrophoresis (NAGE). Aliquots of the gradient shown in A were run in 1% agarose gels; they were either stained with CB or their gel content was blotted onto polyvinylidene difluoride (PVDF) membranes and detected with the monoclonal antibody 10E11. **C** Electron microscopy. Aliquots of the fusion proteins were negatively stained with uranyl acetate.
(TIF)

Table S1 **Alignment of HVRI sequences used for HCVpp neutralization experiments.**
(PDF)
