# Supplementary figures and images for: Hepatitis C Virus Hypervariable Region 1 Variants Presented on Hepatitis B Virus Capsid-Like Particles Induce Cross-Neutralizing Antibodies
Source: PLoS One. 2014 Jul 11;9(7):e102235. doi: 10.1371/journal.pone.0102235 (PMC4094522; doi:10.1371/journal.pone.0102235)

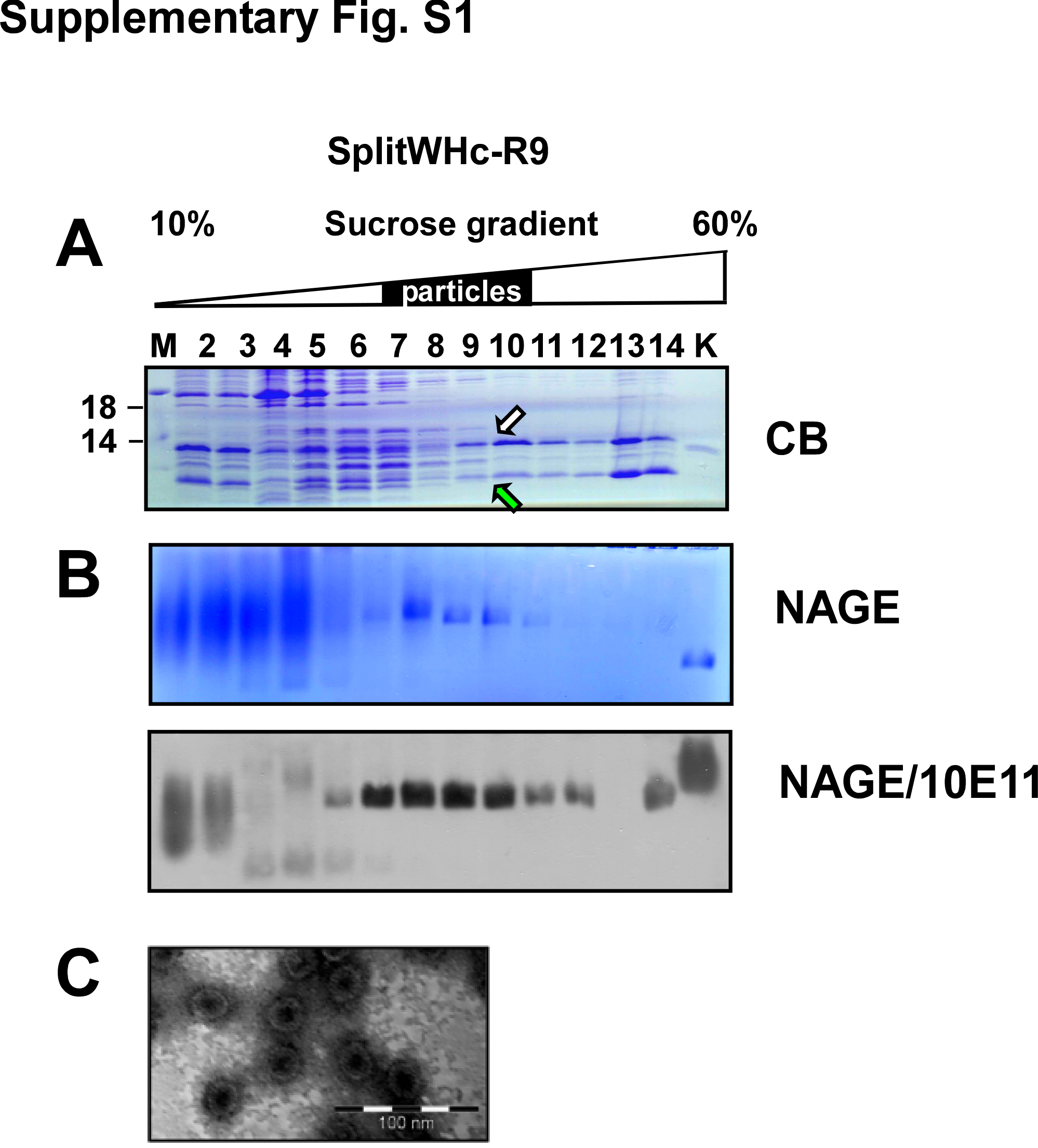

Supplement: Figure S1 — Expression and particle formation of SplitWHC-R9. A Crude lysate from bacteria-expressing SplitWHC-R9 fusion protein was sedimented through a preparative 10% to 60% sucrose step gradient; 14 fractions of 860 µl each were harvested from the top. Aliquots of 8 µl each were analyzed by SDS-PAGE and Coomassie Blue (CB) staining; marker proteins with their molecular masses (in kDa) are indicated on the left. Both fragments, CoreC (arrow up) and CoreN-R9 (arrow down), peaked in the center fractions. B Native agarose gel electrophoresis (NAGE). Aliquots of the gradient shown in A were run in 1% agarose gels; they were either stained with CB or their gel content was blotted onto polyvinylidene difluoride (PVDF) membranes and detected with the monoclonal antibody 10E11. C Electron microscopy. Aliquots of the fusion proteins were negatively stained with uranyl acetate. (TIF) [file pone.0102235.s001.tif]
