# Supplementary material for: Hepatitis C Virus Hypervariable Region 1 Variants Presented on Hepatitis B Virus Capsid-Like Particles Induce Cross-Neutralizing Antibodies
Source: PLoS One. 2014 Jul 11;9(7):e102235. doi: 10.1371/journal.pone.0102235 (PMC4094522; doi:10.1371/journal.pone.0102235)
Supplement: Table S1 — Alignment of HVRI sequences used for HCVpp neutralization experiments. (PDF) [file pone.0102235.s002.pdf]

Name	Sequence	% Neutralization
R9	Q T T V V G G S Q S H T V R G L T S L F S P G A S Q N	-
G31	T - H T - - - - V A R Q - H S - - G - - - - - P Q - K	-
YK5807	V - Y T T - - - - A R H T Q - V A - F - T - - P A - K	-
YK5829	T - - - S - - H A - Q I T - - V - - F - - - - S A - K	-
1a129	E - H - S - - - V A R - T S R - - N - - - - - - M - -	55
1a31	E - Y T S - - V V A Y G T - A - - G F - - Q - S N - -	47
1a38	G - H - T - - - A G R - T A - I A G - L T Q - - K - S	47,6
1a46	G - Y I S - - - A A R A T S - - V - - L T - - - K - -	47
1b44	S - H - S - - - A A - A A S - - - R F - - - - P - - K	59
1b52	T - Y - T - - A T A R - T S - F A - - - T - - - - - K	63,7
1b58	T - N I M - - - - A R D - S R V - - F - - - - - - - K	73,3
1b20	T - Y T S - - - A A Y A T A - - A - - - T - - - R - -	46
1a09	E - Y - T - - - A G - A A S - - A - - - T T - - K - -	17,6
1a142	R - R - T - - T A G R E T A - F A - I - - R - - K - -	-21
1a154	E - H - T - - N A G R - T A - - V G - L T - - - K - -	32,6
1a154	E - H - T - - N A G R - T A - - V G - L T - - - K - -	13
1a157	E - H - T - - N A G - - - A R - A G - - - - - - K - -	4,0
1a53	N - V L I - - Q A A Y - A S S F - A - L T - - - K - -	-34,8
1a72	E - H - T - - T A G R A A A - - A G - - T Q - - R - S	5,3
1a80	N - V L I - - Q A A Y - A S S F - A - L T - - - K - -	36,2
1b09	S - H - T - - T A - - - T - H F A - - - - S - - - - R	-4,8
1b14	D - H T M - - A A G R D T H K F - - - - - F - - - - -	-4,0
1b14	D - H T M - - A A G R D T H K F - - - - - F - - - - -	32
1b20	E - R T I - S E V G S A T H R F - - V - - - - S R - K	8,1
1b35	A - H T I - - T - G R N T Y - - - T - - - - - S - - R	33,9
1b38	K - Y T T - - A - A - - A - - - - T - - - F - P - - -	33
MLV		-17,2
